# Gray matter integrity predicts white matter network reorganization in multiple sclerosis

**DOI:** 10.1002/hbm.24849

**Published:** 2019-11-05

**Authors:** Angela Radetz, Nabin Koirala, Julia Krämer, Andreas Johnen, Vinzenz Fleischer, Gabriel Gonzalez‐Escamilla, Manuela Cerina, Muthuraman Muthuraman, Sven G. Meuth, Sergiu Groppa

**Affiliations:** ^1^ Department of Neurology and Neuroimaging Center (NIC) of the Focus Program Translational Neuroscience (FTN) University Medical Center of the Johannes Gutenberg University Mainz Mainz Germany; ^2^ Department of Neurology University of Münster Münster Germany

**Keywords:** atrophy, graph theory, multiple sclerosis, network analysis, neuropsychology, structural connectivity, tractography

## Abstract

Multiple sclerosis (MS) is a chronic inflammatory and neurodegenerative disease leading to gray matter atrophy and brain network reconfiguration as a response to increasing tissue damage. We evaluated whether white matter network reconfiguration appears subsequently to gray matter damage, or whether the gray matter degenerates following alterations in white matter networks. MRI data from 83 patients with clinically isolated syndrome and early relapsing–remitting MS were acquired at two time points with a follow‐up after 1 year. White matter network integrity was assessed based on probabilistic tractography performed on diffusion‐weighted data using graph theoretical analyses. We evaluated gray matter integrity by computing cortical thickness and deep gray matter volume in 94 regions at both time points. The thickness of middle temporal cortex and the volume of deep gray matter regions including thalamus, caudate, putamen, and brain stem showed significant atrophy between baseline and follow‐up. White matter network dynamics, as defined by modularity and distance measure changes over time, were predicted by deep gray matter volume of the atrophying anatomical structures. Initial white matter network properties, on the other hand, did not predict atrophy. Furthermore, gray matter integrity at baseline significantly predicted physical disability at 1‐year follow‐up. In a sub‐analysis, deep gray matter volume was significantly related to cognitive performance at baseline. Hence, we postulate that atrophy of deep gray matter structures drives the adaptation of white matter networks. Moreover, deep gray matter volumes are highly predictive for disability progression and cognitive performance.

**Abbreviations**
CI: confidence interval
CIS: clinically isolated syndrome
DWI: diffusion‐weighted images
EDSS: Expanded Disability Status Scale
FDR: false discovery rate
FOV: field of view
FWHM: full width at half maximum
MP‐RAGE: magnetization prepared rapid gradient echo
MRI: magnetic resonance imaging
MS: multiple sclerosis
MT: middle temporal
OR: odds ratio
ROI: region of interest
RRMS: relapsing–remitting multiple sclerosis
*SD*: standard deviation
SDMT: Symbol Digit Modalities Test
TE: echo time
TI: inversion time
TMT‐A: Trail Making Test part A
TMT‐B: Trail Making Test part B
TR: repetition time
TSE: turbo spin‐echo

## 1 INTRODUCTION

The conception of the human brain as a complex network includes elements that integrate neural information (gray matter), as well as their connecting pathways (white matter). In multiple sclerosis (MS), a chronic inflammatory autoimmune disease, this network is reconfigured due to demyelinating and inflammatory processes, which strongly alter neural processing (Fleischer, Radetz, et al., 2019). Ultimately, consequences include physical disability and cognitive impairment in domains such as processing speed, episodic memory, and executive functions (Johnen et al., 2017).

Advanced imaging techniques allow investigation of both the integrity of white and gray matter in order to evaluate disease progression (Fleischer et al., 2017; Fleischer, Radetz, et al., 2019; Muthuraman et al., 2016). By means of magnetic resonance imaging (MRI), a decrease of cortical thickness has been observed in the posterior cortices, temporal lobe, and precentral gyrus with progressing MS (Eshaghi et al., 2018; Steenwijk et al., 2016). Subcortical volume mainly decreased in deep gray matter structures including thalamus, caudate, and brain stem, as well as cerebellar regions (Deppe et al., 2016; Eshaghi et al., 2018) in early stages of MS. Atrophy of deep gray matter nuclei is strongly related to cognitive impairment in MS (Damjanovic et al., 2017). In particular, thalamic atrophy is predictive of cognitive decline (Papathanasiou et al., 2015; Štecková et al., 2014) and correlates with physical disability (Deppe et al., 2016). Gray matter atrophy, measured with MRI, reflects neuronal and axonal loss (Popescu et al., 2015). However, although addressed by several studies (Bergsland et al., 2015; Bodini et al., 2009; Henry et al., 2009), it is not yet clear whether it is primarily driven by gray matter neurodegeneration, or a consequence of white matter damage (Steenwijk et al., 2015).

Diffusion‐weighted imaging allows to evaluate white matter microstructural integrity, as more intact myelination leads to more pronounced directional water flow in nerve tracts (Basser, Mattiello, & LeBihan, 1994), which can be quantified using MRI. Connections between brain regions are inferable with methods such as probabilistic tractography (Basser, Pajevic, Pierpaoli, Duda, & Aldroubi, 2000). The impact of pathological processes on both white, as well as gray matter network configuration and efficiency in MS can then be assessed using graph theory (Fleischer, Radetz, et al., 2019).

Applying this approach, patients with relapsing–remitting MS (RRMS) could be distinguished from those with clinically isolated syndrome (CIS) based on stronger decomposition of their network into distinct modules (Muthuraman et al., 2016). Lower global and local network efficiency within white matter networks of RRMS patients was significantly associated with clinical disability, disease duration, and white matter lesion load (Shu et al., 2011). Further, lower global and local efficiency, as well as clustering, were related to worse attention and information processing capacities (Shu et al., 2016). In a further study, a decrease in clustering and global efficiency was observed in patients with RRMS compared to healthy controls, while the distance measure path length was increased (Llufriu et al., 2017). As a first step to investigate network alterations in relation to disease progression, groups of patients with different disease durations were investigated (Fleischer et al., 2017). Modularity and clustering coefficient appeared to increase in the first year after disease onset and subsequently decrease, suggesting an initial compensatory response that cannot be obtained with disease progression (Fleischer et al., 2017).

In a recent approach, information from gray matter and white matter networks was combined in order to evaluate which MRI parameters best explain cognitive performance (Charalambous et al., 2019). While controlling for age, gender, lesion load, and brain volume, the authors found that global efficiency significantly explained additional variance of processing speed and clinical disability scores.

Firstly, we aimed to identify regions of pronounced gray matter atrophy over the one‐year study period. We were then interested in how neurodegenerative processes in these regions relate to white matter network properties of modularization, characteristics relevant for local information processing, and overall network distance change (i.e., emerging disconnection) over time. These measures have previously proven to be highly relevant markers of network functioning in MS and other neurodegenerative disorders (Fleischer et al., 2017; Koirala et al., 2018). However, it is still an unanswered question whether gray matter damage or white matter network reorganization occurs first. Hence, we examined whether gray matter integrity is predictive of white matter network reconfigurations, or whether white matter network parameters can predict gray matter integrity variations, that is, gray matter atrophy. Lastly, we assessed which of the gray and white matter integrity parameters best predict physical disability and cognitive performance.

## 2 METHODS

### 2.1 Participants

We analyzed data of 83 patients (58 female, mean age = 35.33 years, *SD* = 10.84 years) with early RRMS (*n* = 61) and CIS (*n* = 22), who were diagnosed according to the revised McDonald criteria 2010 (Polman et al., 2011) from the Clinic of Neurology. CIS and RRMS were classed as early if the disease duration (defined as time between the manifestation of a patient's first symptoms and the date of the first MRI examination) was ≤3 years. Table 1 provides descriptive demographic details of all patients and an overview of the distribution of the patients' ongoing therapies at the time of MRI examinations. The following exclusion criteria were applied for all patients: any preexisting medical condition known to be associated with brain pathology; pregnancy; previous or current addiction to substances; relapses or systemic therapy with steroids (intravenous, intrathecal, or oral) within the month before the MRI examination; a history of additional neurological or psychiatric disorders. All patients were measured at two time points (mean = 1.12, *SD* = 0.48 years apart) in the same MRI scanner. At the time of both MRI examinations, all patients were physically and neurologically examined. The same specially trained and certified neurologist (JK) assessed Expanded Disability Status Scale (EDSS) scores in all patients (Kurtzke, 1983). From our cohort, 15 patients experienced one relapse between the two measurements, and one patient experienced two. Relapses occurred at least 1 month, and maximally 11 months after the first MRI measurement (mean = 4.5, *SD* = 3.5 months). Relative to the follow‐up measurement, relapses occurred at least 1 month, and maximally 18 months before (mean = 10.8, *SD* = 5 months).

**Table 1:** Demographic, clinical, and neuropsychological data and overview of the distribution of therapies for all patients at the time of MRI examinations

Gender (female/male)	58/25	
Age in years (mean ± *SD*) at *t*_1_	35.33 ± 10.84	
Subtype of multiple sclerosis (RRMS/CIS) at *t*_1_	61 / 22	
Number of patients with relapse between *t*_1_ and *t*_2_	16	
Disease duration in years (mean ± *SD*)	***t***_**1**_	***t***_**2**_
	0.69 ± 0.70	1.82 ± 0.91
EDSS (median [interquartile range])	***t***_**1**_	***t***_**2**_
	1.0 [1.0]	1.5 [1.0]
SDMT raw scores (mean ± *SD*) at *t*_1_	49.05 ± 11.94	
TMT‐A raw scores (mean ± *SD*) at *t*_1_	27.37 ± 10.10	
TMT‐B raw scores (mean ± *SD*) at *t*_1_	71.10 ± 40.10	
Disease modifying therapies (number of patients)	***t***_**1**_	***t***_**2**_
Beta interferons	18	21
Natalizumab	9	13
Glatiramer acetate	8	9
Fingolimod	8	9
Dimethyl fumarate	4	10
Alemtuzumab	2	9
Teriflunomide	2	3
No therapy	32	9

Abbreviations: EDSS, expanded disability status scale; SD, standard deviation; SDMT, Symbol Digit Modalities Test; *t*_1_, baseline MRI; *t*_2_, follow‐up MRI; TMT‐A, Trail Making Test part A; TMT‐B, Trail Making Test part B.

The study was approved by the interdisciplinary ethics committee of the University of Münster and the Physicians' Chamber of Westphalia‐Lippe (Ärztekammer Westfalen‐Lippe, 2010‐378‐b‐S, 2017‐754‐f‐S). Patients were informed of the study content in both oral and written form. All subjects gave written informed consent before participating in this study.

### 2.2 Neuropsychological assessment

Twenty (12 RRMS, 8 CIS) of 83 patients underwent neuropsychological cognitive assessment using a comprehensive test battery. An experienced neuropsychologist (AJ) conducted all neuropsychological tests. The test battery was composed of official German versions of standardized neuropsychological paper–pencil and computerized tests widely established for the use in patients with early MS. The Symbol Digit Modalities Test (SDMT), the Trail Making Test part A (TMT‐A) and part b (TMT‐B) were employed within the year of the first MRI measurement (time interval between neuropsychological assessment and MRI was mean = 2.4, *SD* = 3.4 months). The SDMT is a short screening test for information processing speed in which numbers have to be assigned to abstract symbols as fast as possible. Visual attention and task switching abilities were assessed by the TMT, in which patients are instructed to connect a set of dots with numbers and letters according to specific rules. For all analyses including cognitive variables, we used percentile rank scores obtained by comparing patient's raw scores with test‐specific normative data stratified for age and education. Average raw scores and *SD*s of all three assessments are depicted in Table 1.

### 2.3 MRI

Patients were scanned in a 3T MAGNETOM Prisma^fit^ MRI scanner using a 20‐channel (matrix) head coil (Siemens Healthcare, Erlangen, Germany). Employing the same MRI parameters and protocol for each patient at both time points, we obtained a 3D isotropic T1‐weighted magnetization prepared rapid gradient echo (MP‐RAGE) sequence (echo time [TE] = 2.28 ms, inversion time [TI] = 900 ms, repetition time [TR] = 2,130 ms, flip angle = 8°, field of view [FOV] = 256 × 256 mm^2^, matrix size = 256 × 256, slice thickness = 1 mm, voxel size = 1 × 1 × 1 mm^3^). Further, diffusion‐weighted images were obtained (DWI, 72 axial slices, TE = 69 ms, TR = 8,400 ms, flip angle = 90°, matrix size = 128 × 128, slice thickness = 1.8 mm, voxel size = 1.8 × 1.8 × 1.8 mm^3^). Sixty volumes were acquired with diffusion weighting (*b* = 1,000 s/mm^2^) and 12 were nondiffusion‐weighted (*b* = 0 s/mm^2^). For information about lesion volume, a sagittal 3D turbo spin‐echo (TSE) fluid attenuated inversion recovery (FLAIR) sequence was recorded (TE = 389, TI = 1,800, TR = 5,000, FOV = 256 x 256, slice thickness = 1 mm, voxel size = 1 × 1 × 1 mm^3^, no gap, slice order interlaced).

### 2.4 Quantification of lesion volume

Lesion volume was estimated using the lesion growth algorithm (Schmidt et al., 2012) as implemented in the lesion segmentation toolbox (LST) version 2.0.15 (https://www.applied-statistics.de/lst.html) of the statistical parametric mapping (Penny & Henson, 2006) software (SPM12). T1‐weighted volumes were segmented into cerebrospinal fluid, gray matter, and white matter maps. TSE‐FLAIR images were coregistered to the T1‐weighted volumes and information about the tissue classes used to compute lesion belief maps. These maps were thresholded with an initial threshold of κ *=* 0.1, which was chosen as the optimal value after visual inspection. A binary lesion map was obtained by growing lesions along voxels that appeared hyperintense in the TSE‐FLAIR images.

### 2.5 Data preprocessing, network reconstruction, and network parameter computation

**Figure 1:**
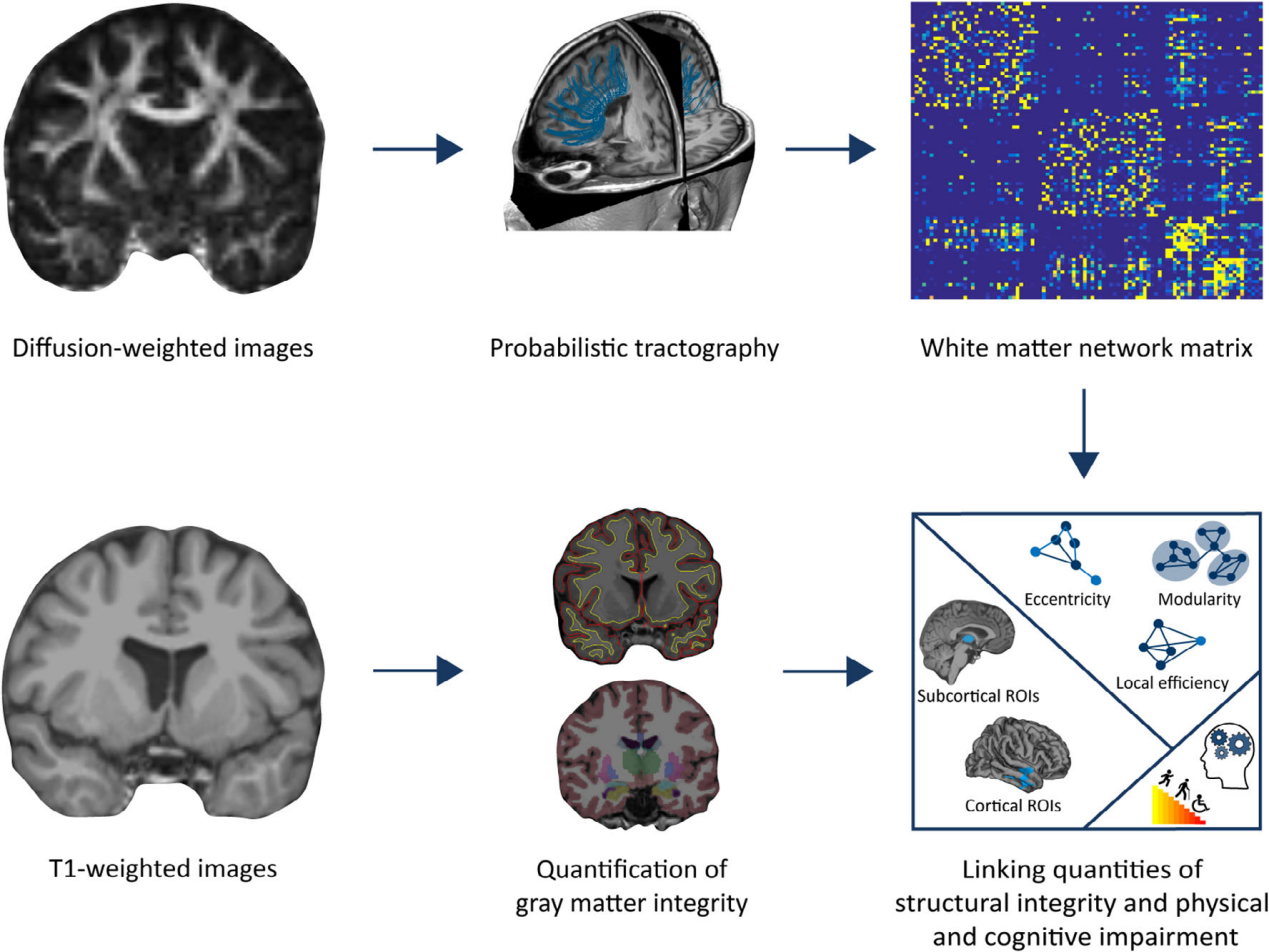
Overview of the processing pipeline. Based on diffusion‐weighted images, probabilistic tractography was conducted. This yielded a white matter network connectivity matrix, from which we computed network measures of modularity, distance, and local information transfer. Reconstructed from T1‐weighted images, cortical thickness and deep gray matter volume of regions of interest was estimated. Finally, we set gray matter integrity and white matter network measures into relation with parameters of physical disability and cognitive impairment. Abbreviation: ROI, region of interest

The cortical surface was automatically reconstructed from T1‐weighted images using the longitudinal processing stream (Reuter, Schmansky, Rosas, & Fischl, 2012) of FreeSurfer version 5.3.0 (http://surfer.nmr.mgh.harvard.edu/). Here, an unbiased within‐subject template space and image was created (Reuter & Fischl, 2011) using robust, inverse consistent registration (Reuter, Rosas, & Fischl, 2010). Reliability and statistical power is significantly increased by initializing with common information from this template, while performing processing steps including skull stripping, Talairach transforms, atlas registration, spherical surface maps, and parcellations (Reuter et al., 2012). DWI were preprocessed using FSL version 5.0.9 (https://fsl.fmrib.ox.ac.uk/fsl/), including motion artifact and eddy current correction (diffusion toolbox of FSL). Crossing fiber distributions and the probability of major and secondary fiber directions were computed. In order to track fibers through regions of crossing or complexity, a multi‐fiber model was fit to the diffusion data at each voxel (Behrens et al., 2003). To estimate the probability distribution of connections from each voxel, 5,000 streamlines were drawn. Seed masks were individually defined based on the 68 cortical (Desikan et al., 2006) and 26 deep gray matter regions (Fischl et al., 2002) reconstructed with FreeSurfer. Tracts that passed through at least one other seed mask were retained. The probabilistic tractography matrix was then generated for each subject. Here, each cell represented the number of streamlines passing through any two regions, normalized for the total number of streamlines drawn from a region. We computed graph theoretical measures of network modularity, distance, and local information transfer with the Brain Connectivity Toolbox (Rubinov & Sporns, 2010; https://sites.google.com/site/bctnet/), as these measures have been shown to be clinically relevant (Fleischer et al., 2017; Koirala et al., 2018). In the graph theoretical framework, brain regions are considered as nodes, and their connections as edges (Fornito, Zalesky, & Bullmore, 2016). Modularity is a measure of the degree, to which the network is subdivided into strongly interconnected nodes with few connections to other groups of nodes (Newman, 2006). The eccentricity of a node is the maximum of its finite distances to all other vertices (Sporns, 2003), and we computed the average of all eccentricity values within the network. Local efficiency, computed with Dijkstra's algorithm, is the average of the inverse distance matrix in sub‐clusters and reflects to what extent neighboring regions of a node remain connected after this node is taken out of the network (Latora & Marchiori, 2001). An overview of the processing pipeline is depicted in Figure 1.

### 2.6 Statistical analyses

#### 2.6.1 Analysis 1: Gray matter atrophy

For identification of a sufficiently large number of atrophying cortical and deep gray matter regions, we chose a threshold of *p* < .05 corrected for multiple comparisons. Cortical atrophy surface maps were smoothed with a 10 mm full width at half maximum (FWHM) Gaussian kernel. General linear model results were corrected for multiple comparisons using Monte Carlo simulation implemented in FreeSurfer's Qdec (Hagler Jr, Saygin, & Sereno, 2006), and average thicknesses of vertices belonging to significant clusters were extracted for each subject.

For deep gray matter regions, we extracted average volume values as computed using FreeSurfer at both time points. We identified regions of interest based on the regional deep gray matter atrophy rate ((GM_*t*2_ − GM_*t*1_)/*t*_diff_), with GM_*t*1_ and GM_*t*2_ as the volume values at the time point of baseline (*t*_1_) and follow‐up (*t*_2_) MRI examination, respectively, and *t*_diff_ as the time interval between any two longitudinal measurements. Subsequently, we applied a false discovery rate (FDR)‐correction to all 26 comparisons computed with *t* tests against zero atrophy using MATLAB (MathWorks, Natick, MA) and included significantly atrophied regions in our further analyses. To further resolve, which brain stem regions account for possible observed effects, we applied FreeSurfer's brain stem parcellation algorithm (Iglesias et al., 2015) and extracted volume values for medulla oblongata, midbrain, pons, and superior cerebellar peduncle separately. For further regression analysis, we averaged cortical thickness values, as well as deep gray matter volume values for regions exhibiting significant atrophy.

#### 2.6.2 Analysis 2: Gray matter integrity and white matter networks

The relation between gray matter thickness and volume and white matter network organization was evaluated using stepwise linear regression analysis in IBM SPSS Statistics for Windows (IBM Corp., Armonk, NY).

After delimiting regions exhibiting atrophy over the one‐year period, we used volume and thickness values of those areas at the first time point in order to predict network reconfigurations. Variations in network measures were computed as the rate of change: ([WM_*t*2_ − WM_*t*1_]/*t*_diff_), with WM_*t*1_ and WM_*t*2_ as the network measure values at the time point of baseline and follow‐up MRI examination, respectively, and *t*_diff_ as the time difference between any two longitudinal measurements. Next, we set up a model to predict the observed cortical and deep gray matter atrophy using white matter network properties at *t*_1_ as predictors.

#### 2.6.3 Analysis 3: Structural integrity and physical disability

We computed an ordinal regression to predict physical disability (EDSS score) at *t*_2_. In one model, we entered the network parameters modularity, eccentricity, and local efficiency at *t*_1_ as variables of interest additionally to the control variables in a stepwise manner. In a second model, we entered deep gray matter volume and cortical thickness of atrophying regions at *t*_1_ additionally to control variables again in a stepwise manner.

#### 2.6.4 Analysis 4: Structural integrity and cognitive performance

The relation between gray matter integrity, white matter network configuration, and cognitive performance was investigated using stepwise linear regression analysis. We inserted deep gray matter volume and cortical thickness at *t*_1_ of regions of interest, as well as eccentricity, modularity and local efficiency at *t*_1_ as variables of interest in three separate models each for SDMT, TMT‐A, and TMT‐B as dependent variable.

For all regression analyses, we included age, gender, lesion, and total brain volume as control variables. Variables with *p* < .01 were included in the final models.

## 3 RESULTS

### 3.1 Analysis 1: Gray matter atrophy

**Figure 2:**
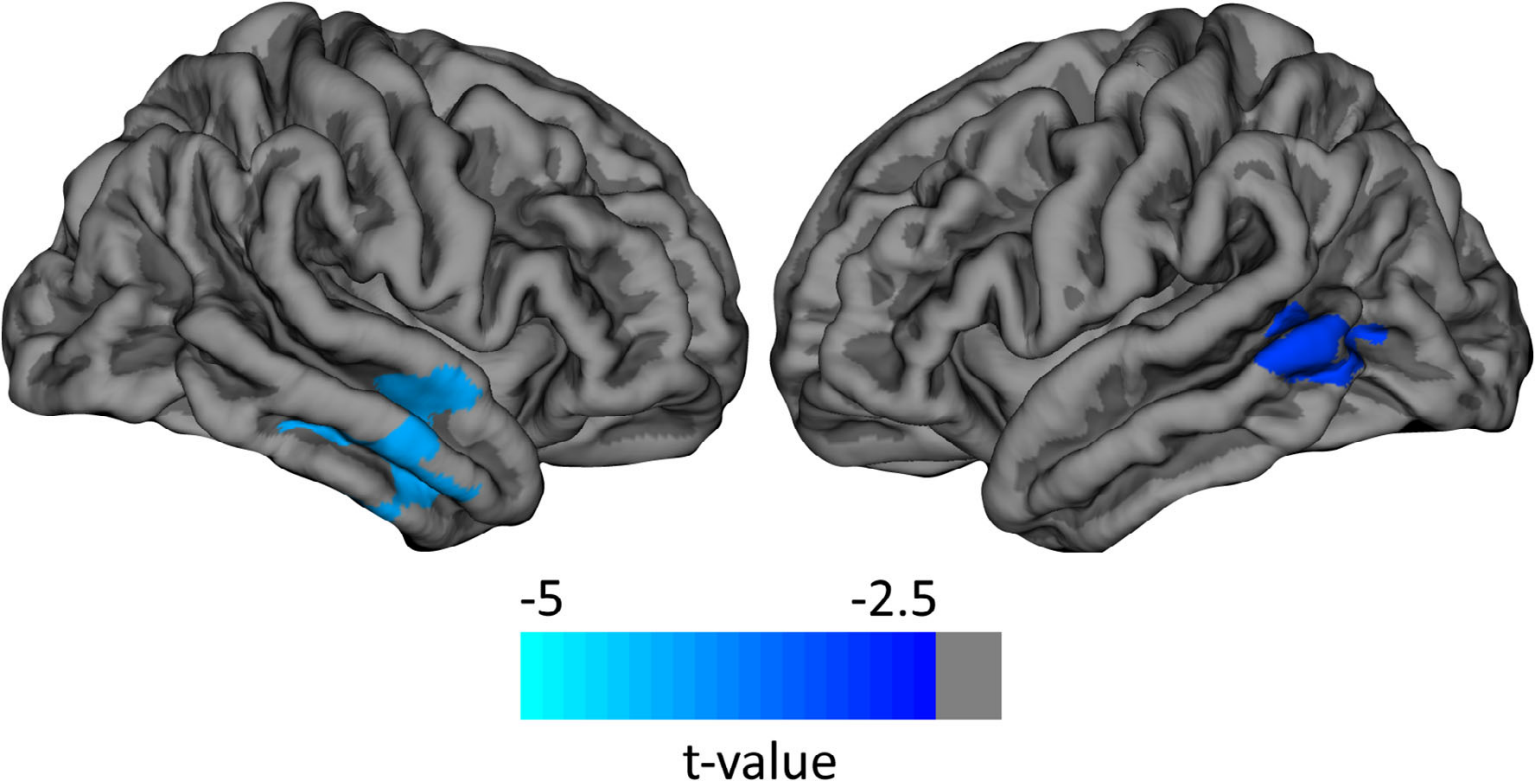
Atrophy *t* values in middle temporal areas. Using FreeSurfer's Qdec, atrophy analysis revealed two clusters in left and right middle temporal areas. Average *t* values of these regions of interest are projected on FreeSurfer's fsaverage template (Fischl, Sereno, Tootell, & Dale, 1999)

**Figure 3:**
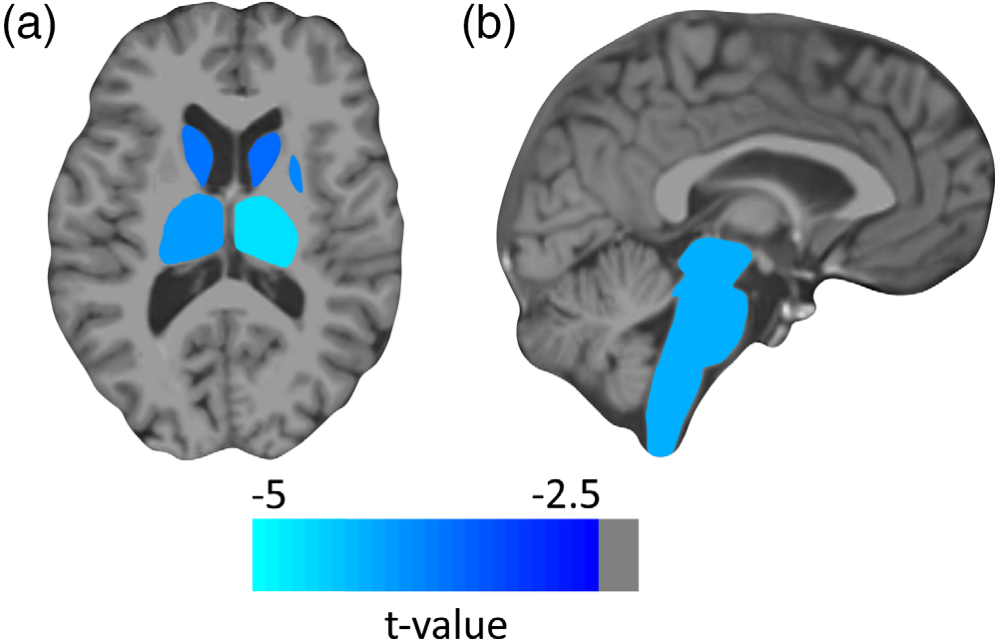
Atrophy *t* values in subcortical areas. *T* values of regional atrophy in deep gray matter regions are depicted on a representative patient's T1‐weighted image. (a) Bilateral thalamus, bilateral caudate, and right putamen. (b) Brain stem

We identified regions of interest by assessing cortical and deep gray matter atrophy over the 1‐year measurement period. Left and right middle temporal (MT) cortices were significantly atrophied (both *p* < .05, Monte Carlo Simulation; Figure 2). Atrophied deep gray matter included bilateral thalamus, caudate, brain stem, and right putamen (*p* < .05, FDR corrected; Figure 3). After further parcellating the brain stem, we detected that the pons mainly accounted for the observed atrophy effects (*p* < .05, FDR corrected, while *p* > .05 for medulla, superior cerebellar peduncle, ventral diencephalon, and midbrain).

### 3.2 Analysis 2: Gray matter integrity and white matter networks

We modeled white matter network reconfigurations regarding modularity, eccentricity, and local efficiency controlling for age, gender, lesion volume, and total brain volume at *t*_1_. Average volume of bilateral thalamus, caudate, brain stem, and right putamen as well as bilateral MT cortex thickness at *t*_1_ as atrophying regions of interest were added in a stepwise linear regression analysis (Table 2). While lesion volume alone accounted for 23% of the variance of eccentricity rate (*p* < .001), 32% of the variance was explained by additionally including the volume of atrophying deep gray matter at *t*_1_ (*p* = .002, Table 2). We performed the same analysis with modularity rate as dependent variable. The best model involved the volume of deep gray matter regions of interest at *t*_1_ as predictor (*p* < .005; Table 2). Cortical thickness did not explain variance of eccentricity or modularity rate. Regarding local efficiency rate, neither deep gray matter volume, nor cortical thickness accounted for variance in addition to the covariates.

**Table 2:** Results of stepwise linear regression analysis of eccentricity and modularity rate

	Adjusted *r* ^2^	Regression coefficient	95% CI	*p* Value
**Eccentricity rate**				
*Model 1*	.226			
Lesion volume		0.290	0.170–0.411	<.001
*Model 2*	.320			
Lesion volume		0.253	0.137–0.369	<.001
Deep gray matter volume		−0.002	−0.003 to −0.001	.002
				
**Modularity rate**				
*Model 1*	.084			
Deep gray matter volume		−1.087 × 10^−5^	−1 × 10^6^ to 1 × 10^−6^	.005

*Note*: Adjusted *r* ^2^ was .233 for the prediction of eccentricity rate (*p* < .001) and .038 for modularity rate (*p* > .05) with the control variables age, gender, lesion, and total brain volume only. Abbreviation: CI, confidence interval.

We next investigated the reverse direction, that is, whether atrophy can be predicted by network parameter values at *t*_1_. No additional variance could be explained by network parameters at *t*_1_ either for deep gray matter, or for cortical atrophy (all *p* > .01).

### 3.3 Analysis 3: Structural integrity and physical disability

Predicting physical disability, the strongest model of the ordinal regression analysis included deep gray matter volume at *t*_1_ in addition to control variables, explaining 17% of the variance of EDSS score at *t*_2_ (*p* = .003, Table 3). No other variable of interest improved the model significantly.

**Table 3:** Results of ordinal regression analysis of EDSS at *t*_2_

	Nagelkerke's *r* ^2^	Parameter estimate	OR	95% CI of OR	*p* Value
**EDSS at** ***t***_**2**_	.166				
Age		0.034	1.035	0.9986–0.9997	n.s.
Gender		−0.272	0.762	0.277–2.092	n.s.
Lesion volume		0.018	1.018	0.973–1.065	n.s.
Brain volume		4.077 × 10^−6^	1 + 4 × 10^−6^	1 + 2 × 10^−7^–1 + 8 × 10^−6^	.04
Deep gray matter volume		−0.001	0.999	0.998–1	.003

*Note*: Nagelkerke's *r* ^2^ was .07 for the control variables age, gender, lesion, and total brain volume only. Abbreviations: CI, confidence interval; OR, odds ratio.

### 3.4 Analysis 4: Structural integrity and cognitive performance

Average scores of neuropsychological test results are depicted in Table 1. Two of three models yielded significant results regarding cognitive tests (Table 4). In addition to the control variables, volume and thickness of atrophying regions at *t*_1_, eccentricity, modularity, and local efficiency at *t*_1_ were also entered into the models. Including lesion volume (*p* < .001), the model explained 38% of the variance of SDMT scores, while no variable of interest explained additional variance. Regarding TMT‐B results, gender alone (*p* < .004) explained 40% of the variance. Adding deep gray matter volume of atrophying regions at *t*_1_ improved the model, accounting for 61% of the variance (*p* < .001 for gender and *p* = .010 for deep gray matter volume). To test whether male patients in the subgroup were more severely affected also regarding physical disability, we descriptively compared EDSS scores between males and females. Male patients (*n* = 10, median = 1.25, interquartile range = 1.5) were more disabled than females (*n* = 10, median = 1, interquartile range = 2).

**Table 4:** Results of stepwise linear regression analysis of neuropsychological scores

	Adjusted *r* ^2^	Regression coefficient	95% CI	*p* Value
**SDMT**				
*Model 1*	.382			
Lesion volume		−1.045	−1.725 to −0.365	.005
				
**TMT‐B**				
*Model 1*	.395			
Gender (male)		−39.244	−64.093 to −14.396	.004
*Model 2*	.605			
Gender (male)		−54.869	−78.544 to −31.194	<.001
Deep gray matter volume		.016	0.004 to 0.027	.010

*Note*: Adjusted *r* ^2^ was .391 for the prediction of SDMT (*p* = .025) and .474 for TMT‐B (*p* = .01) with the control variables age, gender, lesion and total brain volume only. Abbreviation: CI, confidence interval.

## 4 DISCUSSION

In this work, we investigated the relation between gray matter atrophy and white matter network reorganization over a period of 1 year in patients with CIS and early RRMS. Atrophy was mainly observed in deep gray matter regions including the thalamus, caudate, putamen and brain stem, as well as the MT cortex. The longitudinal nature of our study allowed us to track, whether gray matter integrity at baseline predicts white matter network reconfigurations, or whether white matter network parameters at baseline predict gray matter atrophy after 1 year. We chose to study cortical and deep gray matter areas that significantly atrophied within 1 year in our patient cohort as regions of interest. For characterization of white matter networks, we focused on network measures of modularity, local information processing, and distance.

We identified models that predicted each eccentricity and modularity rate. In addition to the control variable lesion volume, volumes of deep gray matter regions of interest at baseline explained further variance in the change of eccentricity, where higher lesion volume and lower deep gray matter volume were associated with an increase in eccentricity over time. Lower deep gray matter volume in regions of interest was also predictive of an increase in modularity and of physical disability at 1‐year follow‐up. In a subsample of the patients, we related MRI variables to neuropsychological parameters. Lower lesion volume was associated with better performance in SDMT. Female gender and higher deep gray matter volume in regions of interest were related to higher TMT‐B ranks.

### 4.1 Gray matter atrophy

Previous works have detected atrophy in deep gray matter regions of patients with MS, including the thalamus, caudate, putamen, and brain stem. These structures have a wide range of functions, including sensory and motor processing, which become restricted in MS due to the accumulating inflammatory and neurodegenerative processes (Deppe et al., 2016; Eshaghi et al., 2018; Steenwijk et al., 2016). We could show that the pons was the structure driving the atrophy effects in the brain stem. The importance of pathological processes in the pons has been outlined previously, as lesions in the pons are predictive of physical disability (Bakshi, Benedict, Bermel, & Jacobs, 2001). Cortically, we observed a significant reduction in thickness of MT areas. This was related to cognitive impairment in a previous study, where the temporal lobe was found to be thinner among all cortical regions in cognitively impaired MS patients than in patients with preserved cognitive function (Tillema et al., 2016). However, here, we did not find an association with neuropsychological scores. This could be due to the small sample size of patients who were neuropsychologically examined or the type of neuropsychological assessments used.

### 4.2 Gray matter integrity and white matter networks

Bringing gray matter integrity and white matter network characteristics together, we found that lower deep gray matter volume was predictive of an increase in the distance measure eccentricity. Higher eccentricity in a network indicates a disruption of information flow, which is rerouted via longer pathways. Deterioration of deep gray matter could cause such a rerouting of information, maintaining information flow, but with reduced efficiency. Equally, higher lesion load causes an increase in eccentricity. Measures of path distance have been shown previously to be increased in patients compared to healthy controls (Llufriu et al., 2017), probably a direct consequence of focal tissue damage. We further showed that increasing modularity can be predicted by lower deep gray matter volume. Previous work from our group revealed that patient networks initially decompose stronger into distinct modules compared to healthy brain networks (Fleischer et al., 2017). This finding raises the question how far variations in modularity and, more generally, functional reorganization of white matter networks are maladaptive or adaptive (Penner & Aktas, 2017; Rocca & Filippi, 2017). A modularity increase could result from a maladaptive breakdown in long‐range connections, dividing the network in a higher number of smaller tightly interconnected units, or from an adaptive compensatory response with stronger intramodular connections (Fleischer et al., 2017). While it was possible to predict changes in white matter network properties based on gray matter atrophy, this was not the case for the reverse direction, such that atrophy was neither predictable in cortical, nor in deep gray matter structures. We hence assume that white matter networks adapt as a response to gray matter depletion.

### 4.3 Structural integrity and physical disability progression

Lower volume of atrophying deep gray matter regions at baseline served as the best predictor for physical disability at 1‐year follow‐up. We, therefore, found that lower deep gray matter volume not only causes adaptations in white matter networks, it is also related to physical disability progression. Conversely, network parameters did not explain any additional variance. This again supports the view that white matter networks initially are less affected by the disease than gray matter integrity. For investigations of patients with longer disease duration, we would expect a relation between white matter network parameters and physical disability as well.

### 4.4 Structural integrity and cognitive performance

We additionally investigated the relation of gray matter integrity and white matter network properties with cognitive performance in a part of the patient sample at baseline. Lower lesion volume was related to an increase in information processing speed, as quantified by SDMT. Male patients scored lower in the TMT‐B, reflecting executive functioning. Gender of healthy subjects does not correlate with TMT scores (Tombaugh, 2004). However, since males are less frequently, but more severely affected by the disease (Greer & McCombe, 2011), an adverse effect of gender on both physical disability and cognitive scores was not unexpected. To further explore this, we additionally compared median EDSS scores between males and females within the subgroup, and observed higher disability in male patients. Our results are in line with prior studies that assessed cognition in MS patients and observed stronger decline in males (Lin et al., 2016; Schoonheim et al., 2012), which moreover was related to EDSS scores (Savettieri et al., 2004). In our cohort, better performance in the TMT‐B was further associated to higher deep gray matter volume in our regions of interest, again in accordance with previous research (Damjanovic et al., 2017; Modica et al., 2016; Schoonheim et al., 2012). The less complex TMT‐A is potentially too insensitive to reflect initial variations in gray matter integrity or network configurations but might be more suitable to distinguish patients of different disease stages. For cognitive performance, we would also expect a relation to white matter network measures after longer disease progression. However, in this retrospective study, only scores from the first measurement time point were available; hence, this needs to be investigated in future work.

### 4.5 Limitations and prospect

Our patient cohort was rather homogeneously mild impaired regarding physical disability and cognitive impairment. Since we only included patients with short disease duration, this limitation is inherently linked to our study design. Therefore, care must be taken when attempting to extrapolate the current results to more severely affected patients. Future studies assessing the relation between gray matter and white matter network integrity including patients with stronger impairment, would thus be of relevance. In addition, the homogeneously distributed physical impairment of our patient group did now allow investigating more specific questions related to the distinct functional systems assessed by the EDSS. In order to track ongoing functional reorganization more closely, longitudinal studies should be conducted with multiple measurement time points. However, this is restricted by repetition effects in the neuropsychological assessments. Further, it would be very informative to consider not only white matter, but also gray matter network reconfigurations longitudinally (Fleischer, Koirala, et al., 2019). Such longitudinal studies including sensitive markers of pathology are rare, however, are increasingly needed for early predictions of the clinical outcome. A complicating factor in MS research is the heterogeneity of patient samples. The disease and its manifestation in the central nervous system is very heterogeneous, and additional variables such as age, gender, treatment, relapse rate, and numerous other parameters are challenging to account for in analyses. In our study, we only considered early stage MS patients; however, it would be interesting to investigate other disease types. For example, more severe cognitive impairment has been observed in MS patients with the primary progressive type (Johnen et al., 2017), while only a few studies have analyzed underlying network reorganization processes (Tur et al., 2019). Considering the variety of confounding variables, analyses would be most informative if they were based on large patient cohorts, for example, from multicenter studies, allowing several sub‐analyses by controlling different influences.

### 4.6 Conclusion

Our results suggest (I) that neurodegeneration predominantly appears in deep gray matter regions after disease onset, and (II) that white matter network reconfiguration occurs as a response to deep gray matter pathology in patients with CIS and early RRMS. Physical disability progression and cognitive deterioration appear to depend on deep gray matter atrophy and the capacity of the network to adaptively reconfigure and maintain information processing efficiency.

## CONFLICT OF INTERESTS

Prof. S. G. Meuth has received honoraria for lecturing, travel expenses for attending meetings, and financial research support from Almirall, Amicus Therapeutics GmbH Deutschland, Bayer Health Care, Biogen, Celgene, Diamed, Genzyme, MedDay Pharmaceuticals, Merck Serono, Novartis, Novo Nordisk, ONO Pharma, Roche, Sanofi‐Aventis, Chugai Pharma, QuintilesIMS und Teva. Dr. J. Krämer received honoraria for lecturing from Biogen, Novartis, Mylan, and Teva, and financial research support from Sanofi Genzyme.

## Data Availability

The data that support the findings of this study are available from the corresponding author upon reasonable request.
